# Mechanism of Butyrate Stimulation of Triglyceride Storage and Adipokine Expression during Adipogenic Differentiation of Porcine Stromovascular Cells

**DOI:** 10.1371/journal.pone.0145940

**Published:** 2015-12-29

**Authors:** Hui Yan, Kolapo M. Ajuwon

**Affiliations:** Department of Animal Sciences, Purdue University, West Lafayette, Indiana, 47907–2054, United States of America; Southern Illinois University School of Medicine, UNITED STATES

## Abstract

Short chain fatty acids (SCFA), products of microbial fermentation of dietary fiber, exert multiple metabolic effects in cells. Previously, we had demonstrated that soluble fiber influenced fat mass accumulation, gut microbial community structure and SCFA production in pigs. The current study was designed to identify effects of SCFA treatment during adipogenic differentiation of porcine stromovascular cells on lipid metabolism and adipokine expression. Differentiating cells were treated with varying concentrations of butyrate. Results show that butyrate treatment enhanced adipogenesis and lipid accumulation, perhaps through upregulation of glucose uptake and *de novo* lipogenesis and other mechanisms that include induction of SREBP-1c, C/EBPα/β, GLUT4, LPL, PPARγ, GPAT4, DGAT1 and DGAT2 expression. In addition, butyrate induced adiponectin expression, resulting in activation of downstream target genes, such as AMPK and AKT. Activation of AMPK by butyrate led to phosphorylation of ACC. Although increased ACO gene expression was seen with butyrate treatment, experiments with the peroxisomal fatty acid inhibitor, thioridazine, suggest that butyrate may have an inhibitory effect on peroxisomal fatty acid oxidation. Our studies also provide evidence that butyrate may inhibit lipolysis, perhaps in an FFAR3-dependent manner. Therefore, this study presents a novel paradigm for butyrate action in adipocytes and shows that adipocytes are capable of utilizing butyrate, leading to increased expression of adiponectin for enhanced glucose uptake and improved insulin sensitivity.

## Introduction

Metabolic syndrome is a cluster of risk factors which include obesity, insulin resistance or type II diabetes, dyslipidemia, hypertension and cardiovascular disease (CVD) [1]. Dietary fiber has a potential to counteract metabolic syndrome phenotype, because it has been reported to lead to decreased fat accumulation and increased insulin sensitivity in humans and animals [2, 3]. Studies also suggest that dietary fiber functions through mechanisms that include alteration of gut microbial community structure in the large intestine [2]. We previously demonstrated [3], that soluble fiber, such as inulin, alleviated high fat diet-induced fat mass accumulation and altered gut microbial structure in pigs. Alteration in gut microbial community structure is strongly associated with changes in short chain fatty acid (SCFA) concentrations in the large intestine [3].

Short-chain fatty acids contain about 1–6 carbon atoms, and are produced by microbial fermentation in the hind gut, especially in the cecum and colon. Their total concentrations can reach up to 100 mM in these hind gut sections [3, 4]. These SCFAs have earlier been associated with alleviation of the metabolic syndrome phenotype [5]. The main colonic and cecal SCFAs in humans and swine are acetate, propionate and butyrate which make up to 95% of total SCFA [3, 6]. In the past few years, several reports have shown that SCFAs play key roles in regulating energy expenditure and insulin sensitivity [7, 8]. However, studies in non-rodent live animal and *in vitro* cell culture models are limited. In this report, we have used porcine adipocytes as our experimental model. This is because the pig is an established biomedical model for humans [9], and studies in pig adipocytes are likely to be relevant to human adipose tissue metabolism.

In addition, there is limited information on the mechanism of chronic effects of SCFA on white adipocyte differentiation, lipid storage (accumulation and release), insulin sensitivity, and adipokine production. Determining the effects of SCFA on white adipocytes is especially critical because of the importance of white adipose tissue as a regulator of whole body energy homeostasis [10].

In order to expand our understanding of mechanism of SCFA effects on metabolism, we have conducted a series of *in vitro* experiments to examine the chronic effects of different SCFAs on lipid metabolism, specifically focusing on their effects on molecular markers of insulin sensitivity, lipogenesis and adipokine expression in pig adipocytes. We hereby provide evidence that SCFAs, especially butyrate, have direct metabolic effects on the adipocyte and that they may mediate some of the metabolic effects of dietary fiber.

## Materials and Methods

### Cell culture and preadipocyte differentiation

The Purdue Animal Care and Use Committee (PACUC) approved all animal care and use procedures used in this study. Piglets (< 7days old) were euthanized with intramuscular injection of atropine, tiletamine-zolazepam, and xylazine followed by pneumothorax and cardiectomy or by CO_2_ exposure followed by severance of the jugular vein and exsanguination. Piglets were confirmed dead before adipose tissue was obtained. Preadipocytes were isolated from subcutaneous adipose through collagenase (Sigma-Aldrich, St. Louis, MO, USA) digestion, filtration and centrifugation. Cells from different piglets were used separately. Briefly, cells were cultured at 37°C under 5% CO_2_ in 24-well tissue culture plates (BD Falcon, Franklin Lakes, NJ, USA). Cells were grown in Dulbecco’s modified Eagle’s medium/F12 (DMEM/F12; Sigma-Aldrich, St. Louis, MO, USA) with 10% fetal bovine serum (FBS; Mediatech. Inc., Manassas, VA, USA) supplemented with 1% penicillin-streptomycin mixture (Mediatech. Inc., Manassas, VA, USA). At 2 days post-confluence (day 0), cells were induced to differentiate with DMEM/F12 with 10% fetal bovine serum, 1.7 μM insulin, 100 nM dexamethasone, 1 nM triiodothyronine (T_3_), 33μM biotin, 17μM pantothenic acid and 1 μM rosiglitazone for 9 days. Medium was changed every 3 days and rosiglitazone was removed on day 3. On day 9, most of the cells were fully differentiated to mature adipocytes with visible lipid droplets. Cells were harvested on day 9 post-differentiation for RNA isolation, protein extraction and triglyceride extraction.

### Short chain fatty acids treatment

To determine the effects of chronic exposure of the different SCFAs on adipocyte differentiation and metabolism, preadipocytes were treated with acetate, propionate and butyrate at induction of differentiation. Sodium salts of SCFA (Sigma-Aldrich, St. Louis, MO, USA) were dissolved in DMEM/F12 to make the SCFA working solution. Media was supplemented with SCFAs throughout the differentiation period (day 0 to 9). Different concentrations of each SCFA were used, ranging from 0 to 1500 μM. Cells were harvested on day 9 of differentiation for RNA and protein extraction to determine effects of SCFAs on adipocyte differentiation and metabolic markers using real-time PCR and western blot analysis of selected genes.

### Glucose uptake and *de novo* lipogenesis assays

Preadipocytes were differentiated into adipocytes for 8 days in 24-well plates with or without butyrate. Before the glucose uptake and lipogenesis assays, cells were incubated overnight in DMEM/F12 containing 0.1% fatty acid free BSA without insulin. For glucose uptake assay, cells were washed twice with PBS and then incubated with PBS in the absence (basal) or presence of 17μM insulin for 30 min. Cells were then incubated in PBS containing 50 μM unlabeled 2-deoxy-D-glucose (Sigma-Aldrich, St. Louis, MO, USA) and 0.5 μCi/mL 2-deoxy-D-[^14^C]-glucose (American Radiolabeled Chemicals, St. Louis, MO) for additional 10 min. Non-specific uptake of 2-deoxy-D-[^14^C]-glucose was determined in the presence of 10 μM cytochalasin B (Cayman, Ann Arbor, MI, USA). Cells were then washed twice with PBS and lysed with 0.05N NaOH. Cell lysates were transferred into scintillation liquid for counting. For lipogenesis assay, cells were washed twice with PBS and incubated in DMEM/F12 containing 0.1% fatty acid free BSA and 0.5 μCi/mL D-[^14^C]-glucose in the absence or presence of 17μM insulin for 2 h. Cells were then washed twice with PBS and lysed with 0.1N HCl. Total lipids were extracted in 2:1 chloroform-methanol (v/v). The chloroform phase was transferred into scintillation liquid for counting. 2-deoxy-D-[^14^C]-glucose or D-[^14^C]-glucose counts were measured as disintegrations per minute (DPM) and normalized to sample protein concentrations measured with the BCA assay.

### Interaction between butyrate and β-adrenergic agonist on lipolysis

To determine the effects of butyrate on lipolysis, cells were treated with β-adrenergic agonist, isoproterenol (ISO) [11] (Sigma-Aldrich, St. Louis, MO, USA), at 10 μM concentration to induce lipolysis. Isoproterenol was applied to adipocytes treated with or without butyrate for 2 hours before harvesting on day 9 of differentiation. Both cells and culture media were harvested for triglyceride and glycerol measurement.

### Inhibition of peroxisomal and mitochondrial fatty acids oxidation

To determine the effects of fatty acid oxidation on butyrate action, cells were treated with either a mitochondrial β-oxidation inhibitor, etomoxir (ETO) [12, 13], at 1 μM concentration or a peroxisomal β-oxidation inhibitor, thioridazine (TRD) [14, 15], at 10 μM concentration. The combination of etomoxir and thioridazine (TE) was also applied to cells. Inhibitors were from Sigma-Aldrich (St. Louis, MO, USA). Inhibitors were applied in the absent or presence of butyrate at the start of induction of differentiation. Medium was replaced with fresh butyrate and inhibitors during each medium change up to day 9 when cells were harvested for RNA extraction, protein extraction and triglyceride or ATP determination.

### RNA extraction and quantitative real-time PCR analysis

Total RNA was extracted from adipocytes using TRIzol^®^ reagent (Invitrogen, Carlsbad, CA, USA), and then reverse transcribed into complimentary DNA (cDNA) using MMLV reverse transcriptase (Promega, Madison, WI). Quantitation of mRNA expression was performed on the MyiQ real-time PCR detection system (Bio-Rad, Hercules, CA, USA) with the SYBR green RT-PCR mix (Qiagen, Foster City, CA, USA). Carnitine palmitoyl transferase 1-alpha (CPT1α) and acyl-CoA oxidase I (ACO) were analyzed as markers of mitochondrial and peroxisomal β-oxidation, respectively. Fatty acid synthase (FAS) was used as a marker for *de novo* fatty acid synthesis. Peroxisome proliferator activated receptor gamma (PPARγ), CCAAT/enhancer binding protein alpha (C/EBPα), C/EBPβ, retinoid X receptor alpha (RXRα) were selected as markers of adipogenesis. Sterol regulatory element binding protein 1c (SREBP-1c), was used as a marker of both fatty acid synthesis and adipogenesis. Glycerol-3-phosphate acyltransferase 4 (GPAT4), Diacylglycerol O-acyltransferase 1 (DGAT1) and DGAT2 were key markers for triglyceride synthesis. Adiponectin and leptin were used as markers of adipokine expression. Glucose transporter (GLUT) type 4 (GLUT4), lipoprotein lipase (LPL), fatty acid transport protein 4 (FATP4), important proteins in cellular glucose and fatty acids uptake, and fatty acid receptor 2 and 3 (FFAR2 and FFAR3), both G protein coupled receptors for SCFAs, were measured as indicators of transporter expression. Real-time PCR primers for these genes are listed in S1 Table. Abundance of specific mRNA transcripts was expressed relative to 18S rRNA using Pfaffl’s method [16].

### Immunoblotting analysis

Adipocytes were lyzed in 1x RIPA lysis buffer (50 mM Tris-HCl, 15mM NaCl, 0.25% deoxycholic acid, 0.1% Triton X, 10 mM EDTA, 0.01% Na_3_VO_4_) with protease inhibitor cocktail and phosphatase inhibitor cocktail (Sigma-Aldrich, St. Louis, MO, USA). Cell lysates were centrifuged at 12,000 g for 10 min at 4°C and cellular protein was recovered in the supernatant. Medium protein was also collected and concentrated through trichloroacetic acid (TCA) precipitation, and then dissolved in 1x RIPA lysis buffer. Protein concentrations were determined using the bicinchoninic acid (BCA) assay kit (Sigma-Aldrich, St. Louis, MO, USA). Total proteins were separated by SDS-PAGE on 10% resolving gel and transferred to 0.2 μm nitrocellulose membranes (Bio-Rad, Hercules, CA, USA) using semi-dry trans-membrane system (Bio-Rad, Hercules, CA, USA). After blocking membranes in 5% bovine serum albumin (BSA) (3% fat free milk was used for media protein blotting) in TBST (50mM Tris-HCl, 150mM NaCl, 0.1% Tween 20, pH 7.4) for 30 minutes, immunoblotting was performed at 4°C overnight with protein-specific primary antibodies at concentrations recommended by the manufacturers. The following primary antibodies were used: anti-PPARγ, anti-phospho Akt, anti-Akt, anti-phospho AMPKα, anti-AMPKα, anti-phospho ACC, anti-ACC, anti-β-actin (Cell Signaling Technology, Danvers, MA, USA), anti-DGAT1 (Neobiolab, Cambridge MA, USA), anti-DGAT2 (Abcam, Cambridge MA, USA), anti-adiponectin (Xeno Diagnostics, LLC, IN, USA) and anti-albumin (BioVision, Inc., Milpitas, CA, USA). Membranes were incubated with a secondary antibody (anti-rabbit IgG-HRP, anti-rabbit horseradish peroxidase linked IgG) (Cell Signaling Technology, Danvers, MA, USA) at a dilution of 1:20,000. Signals were visualized with a chemiluminescent HRP substrate (Millipore, Billerica, MA, USA) on autoradiographic film (Santa Cruz Biotechnology, Santa Cruz, CA, USA). Densitometric analyses of immunoblotting images were performed using the Kodak EDAS290 image system (Kodak, New Haven, CT, USA).

### Triglyceride and free glycerol determination

Adipocytes were washed with PBS (phosphate buffered saline). Intracellular triglyceride in adipocytes was extracted in 100 μL isopropanol (≥99.5%; Thermo Fisher Scientific Inc., Waltham, MA, USA) for 30 minutes at room temperature with constant agitation. Culture media were collected for free glycerol determination. Intracellular triglyceride and free glycerol concentrations were quantified using the Triglyceride Determination Kit (Sigma-Aldrich, St. Louis, MO, USA) according to the manufacturer’s instructions.

### ATP determination

Adipocytes were first rinsed in PBS and lysed in 1x RIPA lysis buffer with a protease and phosphatase inhibitor cocktails as described above. Cell lysates were centrifuged at 10,000 g for 10 min to remove floating lipids. Cellular ATP was measured in the supernatant using an ATP Determination Kit (Invitrogen, Carlsbad, CA, USA) according to the manufacturer’s instructions. ATP values were normalized to protein concentrations quantified with the BCA assay protocol.

### Statistical analysis

Data analysis was carried out with analysis of variance (ANOVA) using the PROC GLM procedure of SAS version 9.2 (SAS Inst. Inc., Cary, NC, USA). Unpaired *t-*test and Tukey multiple comparison were performed to determine significant differences among treatment means. Data were expressed as mean ± SE. P-values less than 0.05 were defined as statistically significant, whereas P-values between 0.05 and 0.1 were considered as a trend.

## Results

### Effect of SCFAs on expression of markers of metabolism

**Fig 1:**
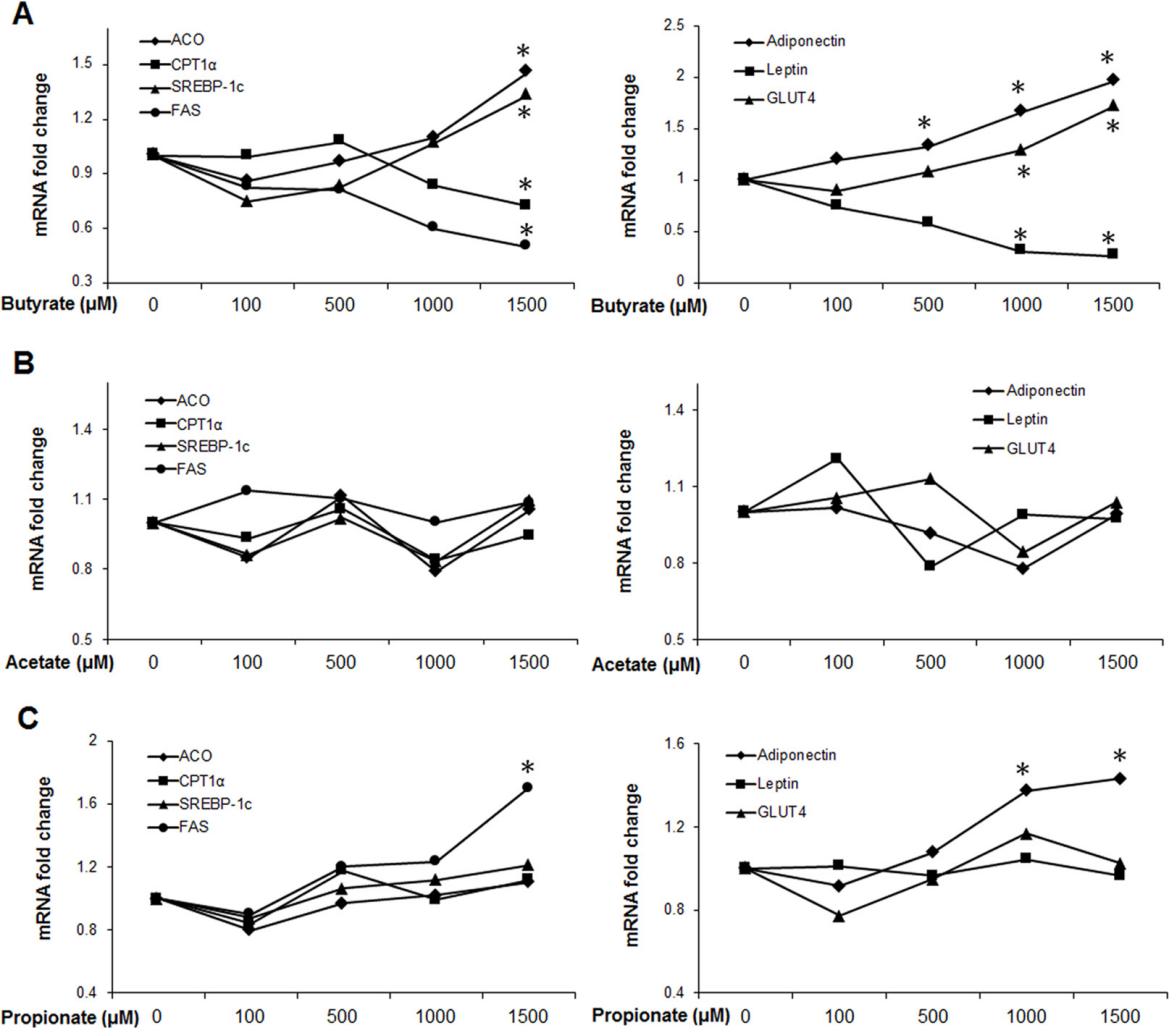
Effects of SCFAs on expression of metabolic and adipokine gene markers. (A) Butyrate supplementation (B) Acetate supplementation (C) Propionate supplementation. Preadipocytes were treated with acetate, propionate or butyrate at induction of differentiation. SCFAs were supplemented in the media throughout the differentiation period (day 0 to 9) at different concentrations, ranging from 0 to 1500 μM. Cells were harvested on day 9 for real-time PCR. Data are means ± SE (n = 12). *P<0.05 vs. 0 μM SCFA supplementation.

To investigate chronic effects of SCFAs on markers of lipid metabolism, cells were exposed to different concentrations of acetate, propionate or butyrate throughout differentiation (day 0–9) at concentrations ranging from 0 to 1500 μM. Butyrate supplementation led to increases in the expression of ACO, SREBP-1c, GLUT4 and adiponectin, but decreased CPT1α and FAS expression, especially at the 1500 μM concentration (P < 0.05) (Fig 1A). Acetate and propionate were also investigated at the same concentrations as butyrate. Although acetate showed no significant effects on these selected gene markers (Fig 1B), propionate significantly increased FAS and adiponectin expression at 1500 μM. Other gene markers were not significantly affected by propionate supplementation (Fig 1C). The implication of the lack of robust response is still unclear, but it might indicate that adipocytes do not have the mechanism to metabolize substantial quantities of acetate and propionate. As butyrate exerted the most pronounced effects relative to acetate and propionate, further experiments were focused on butyrate at the 1500 μM concentration.

### Butyrate supplementation enhances adipogenesis

**Fig 2:**
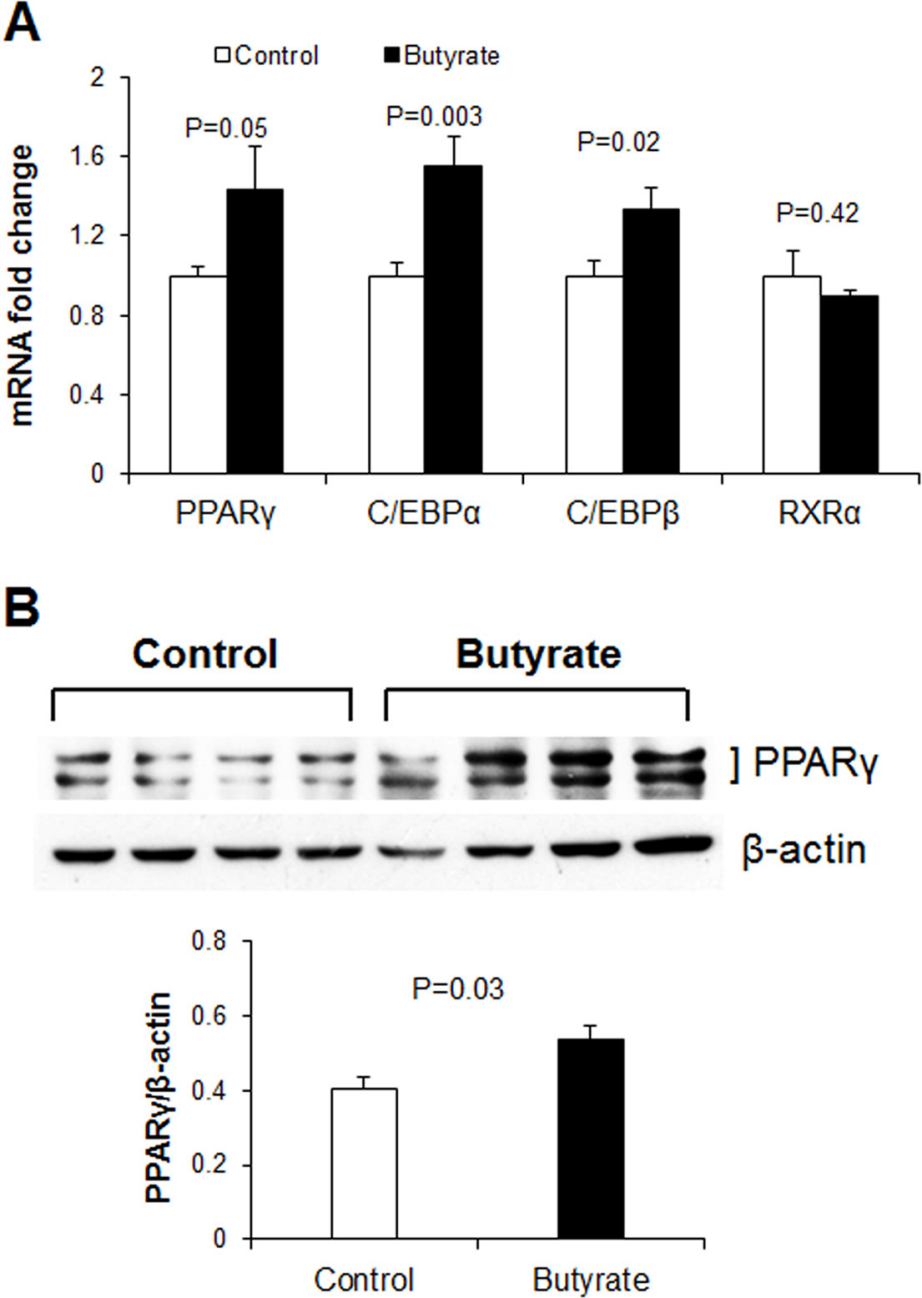
Effects of butyrate on porcine adipocyte differentiation. (A) mRNA expression of adipocyte differentiation markers (B) Representative immunoblotting and quantification analysis of PPARγ abundance in adipocytes. Preadipocytes were treated with butyrate at 1500 μM throughout the differentiation period (day 0 to 9). Cells were harvested on day 9 for real-time PCR and immunoblotting. Data were analyzed by *t* test, and *P* values are indicated on the bars (Control *vs*. Butyrate). Data are means ± SE (A, n = 12; B, n = 4).

Butyrate significantly induced mRNA expression of SREBP-1c, a key regulator for both fatty acid *do novo* synthesis and adipogenesis (Fig 1A). This provides a first indication that butyrate may enhance adipogenesis. We also examined the expression of downstream adipocyte differentiation marker genes such as PPARγ, C/EBPα, C/EBPβ and RXRα [17]. Expression of PPARγ, C/EBPα and C/EBPβ was markedly upregulated in butyrate treated adipocytes compared to control group (Fig 2A), indicating butyrate could indeed enhance adipocyte differentiation. However, expression of RXRα was similar between butyrate and control groups, suggesting that this gene was not a major target of butyrate. A significant increase in PPARγ protein abundance was also observed in butyrate treated adipocytes compared to control (Fig 2B). Taken together, these results suggest that butyrate promotes adipogenesis through enhanced expression of genes that play major roles in adipocyte differentiation.

### Butyrate effect on adipokine expression, insulin sensitivity and AMPK activity

**Fig 3:**
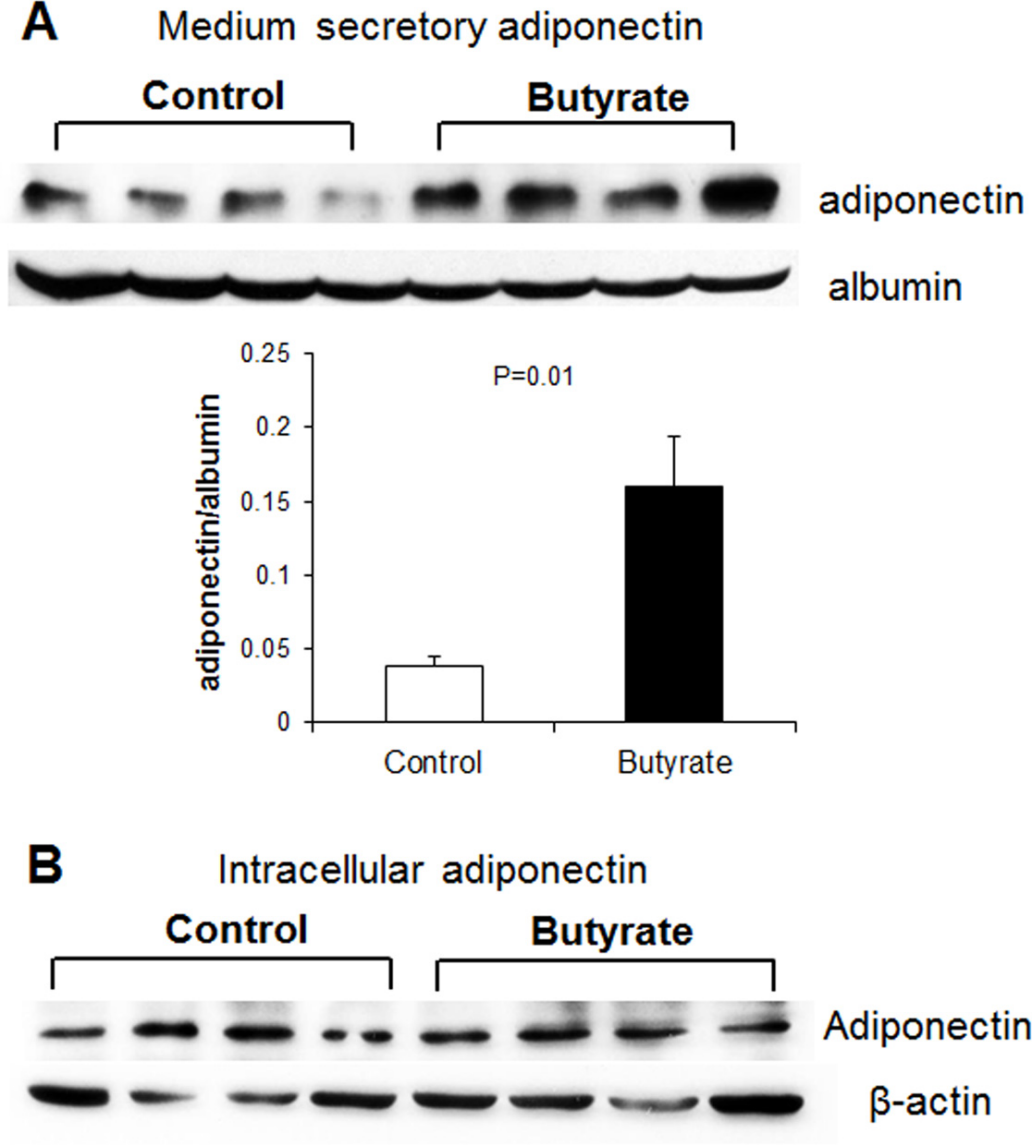
Effect of butyrate on adiponectin protein expression and secretion. (A) Representative immunoblotting and quantification analysis of adiponectin abundance in culture media. (B) Representative immunoblotting analysis of intracellular adiponectin level in the cell lysate. Preadipocytes were treated with butyrate at 1500 μM concentration throughout the differentiation period (day 0 to 9). Both cells and culture media were harvested on day 9 for immunoblotting. Data were analyzed by *t* test, and *P* values were shown on the bars (Control vs. Butyrate). Data are means ± SE (n = 4).

Because adipokines are powerful mediators of metabolism through which SCFAs could exert their effect, we determined the expression of leptin and adiponectin. Butyrate led to increased expression of adiponectin, starting at a much lower concentration threshold (500 μM) (Fig 1A). To verify whether butyrate increased adiponectin protein expression, we also performed immunoblotting for both secretory and intracellular adiponectin. Butyrate supplementation significantly increased secreted adiponectin abundance in the culture media compared to control (Fig 3A), though intracellular adiponectin abundance was similar between butyrate and control groups (Fig 3B).

**Fig 4:**
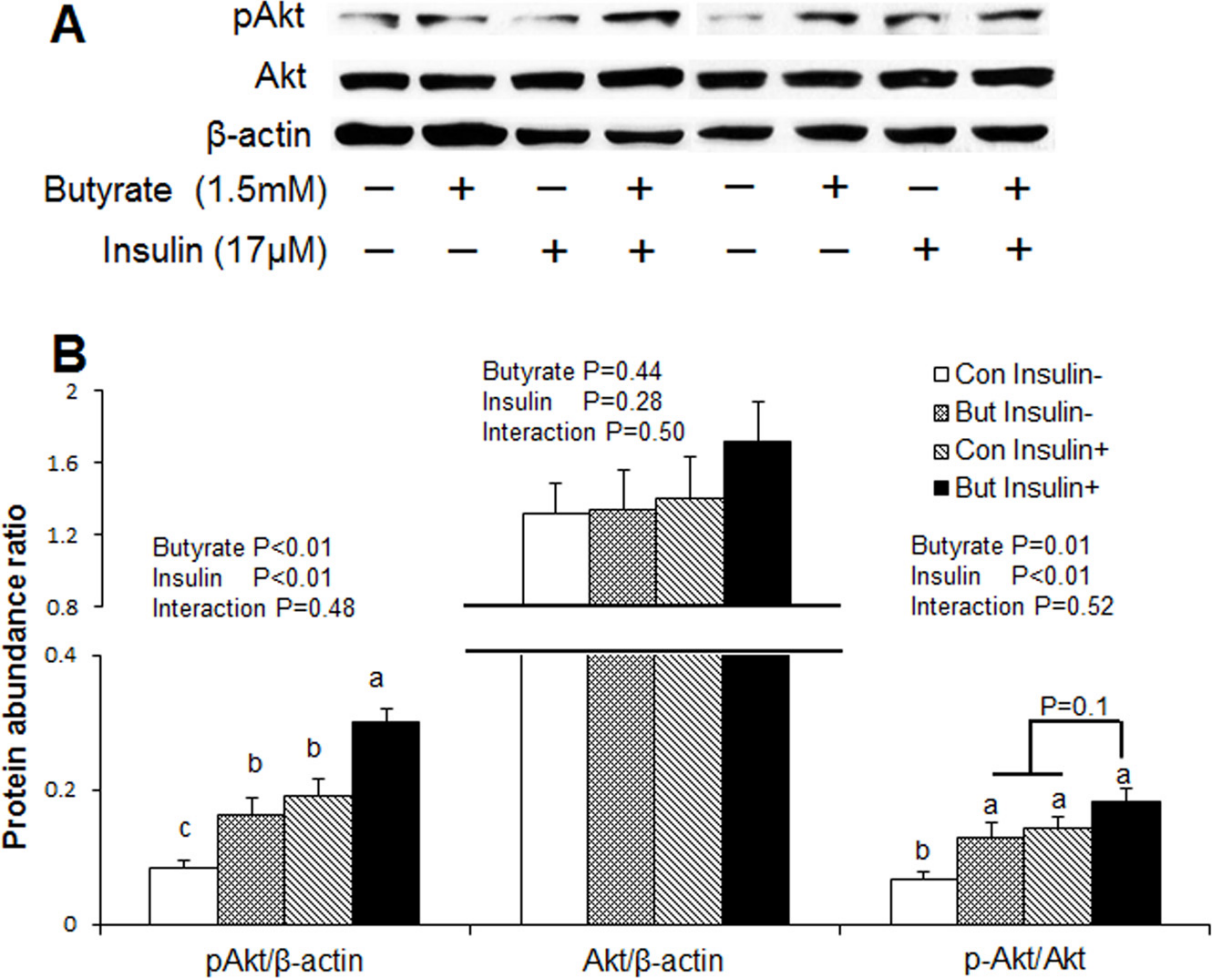
Effects of butyrate on AKT activation in adipocytes. (A) Representative immunoblotting analysis of AKT in adipocytes. (B) Quantification analysis of AKT in adipocytes. Cells were treated with butyrate at 1500 μM throughout the differentiation period (day 0 to 8). On day 8, cells were incubated overnight with DMEM/F12 containing 0.1% BAS in the absence of insulin. On day 9, Butyrate-treated and untreated cells were incubated in the absence or presence of 17 μM insulin for 30 min and then harvested for immunoblotting. Data were analyzed by *two-way* ANOVA with Tukey multiple comparison test. Data are means ± SE (n = 6). Different letters on bars indicate significant differences (*P*<0.05).

**Fig 5:**
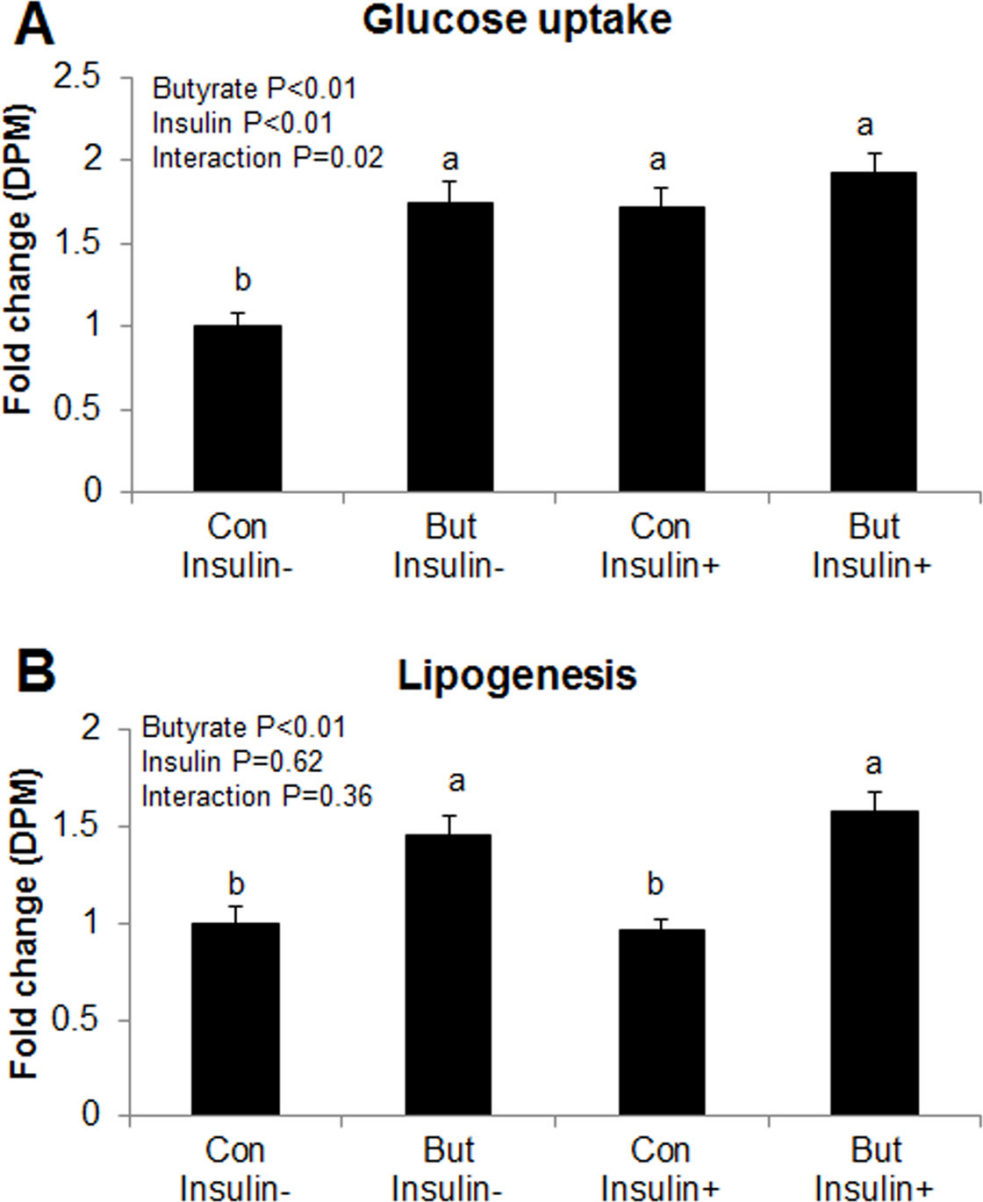
Effect of butyrate on glucose uptake and *de novo* lipogenesis in adipocytes. (A) Glucose uptake assay in adipocytes. (B) *De novo* lipogenesis assay in adipocytes. Cells were treated with butyrate at 1500 μM concentration throughout the differentiation period (day 0 to 8). On day 8, cells were incubated overnight with DMEM/F12 containing 0.1% BSA in the absence of insulin. On day 9, cells were subjected to either glucose uptake or *de novo* lipogenesis assay in the absence or presence of insulin. Data were analyzed by *two-way* ANOVA with Tukey multiple comparison test. Data are means ± SE (n = 20). Different letters on bars indicate significant differences (*P*<0.05).

It is well established that elevated adiponectin level is highly correlated with increased insulin sensitivity [18, 19]. To determine whether butyrate regulates markers of insulin sensitivity, we also conducted immunoblotting for Akt protein, a downstream target of insulin signaling, glucose uptake assay with ^14^C deoxyglucose in control or insulin-stimulated cells in the presence or absence of butyrate. Both butyrate and insulin significantly increased phospho-AKT abundance and phospho-AKT/AKT ratio (P<0.01). Adipocytes treated with a combination of both butyrate and insulin showed highest phospho-AKT abundance (P<0.05) and tendency (P = 0.1) for higher phospho-AKT/AKT ratio, compared to cells treated with either butyrate or insulin alone. However, glucose uptake was lower in the cells under basal condition (untreated) compared to treated cells (Fig 4). Furthermore, we established with glucose uptake assay that both butyrate and insulin significantly increased (P<0.01) glucose uptake (Fig 5A). When combined with the evidence for increased GLUT4 expression (Fig 1A), butyrate showed a potential to increase insulin sensitivity in adipocytes.

**Fig 6:**
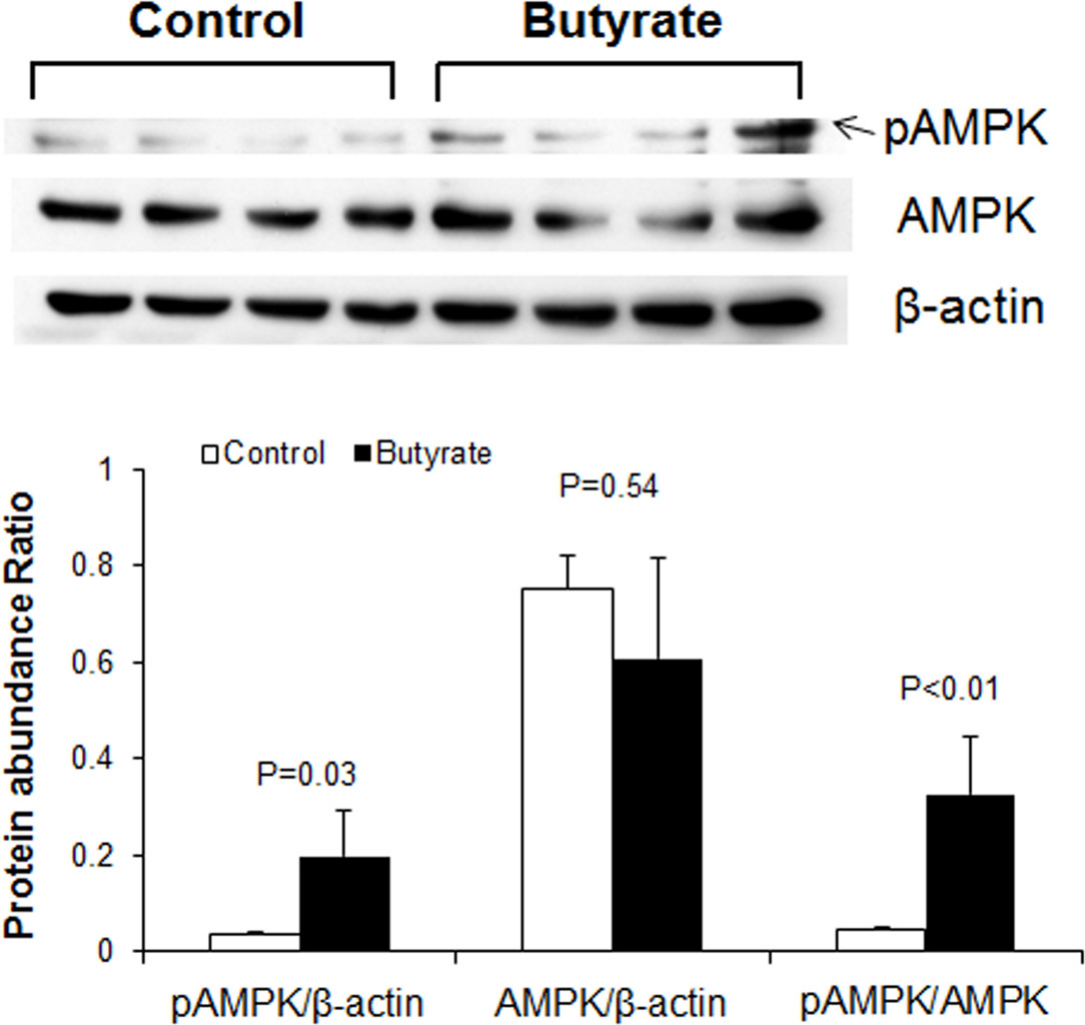
Effects of butyrate on AMPK activation in adipocytes. Preadipocytes were treated with butyrate at 1500 μM throughout the differentiation period (day 0 to 9). Cells were harvested at day 9 for immunoblotting. Data were analyzed by *t* test, and *P* values were shown on the bars (Control vs. Butyrate). Data are means ± SE (n = 4).

Adiponectin activates AMPK in multiple tissues [20–22], and insulin sensitivity is positively correlated to AMPK activity [18, 21]. Therefore, to determine whether butyrate induced AMPK activation, we measured phospho-AMPK and AMPK protein abundance through immunoblotting. Butyrate-treated adipocytes showed significantly higher phospho-AMPK and phospho-AMPK/AMPK ratio compared to control (Fig 6). Taken together, butyrate supplementation increased adiponectin secretion, consequently leading to activation of downstream target, AMPK and AKT pathways.

### Butyrate effect on *de novo* fatty acid synthesis

**Fig 7:**
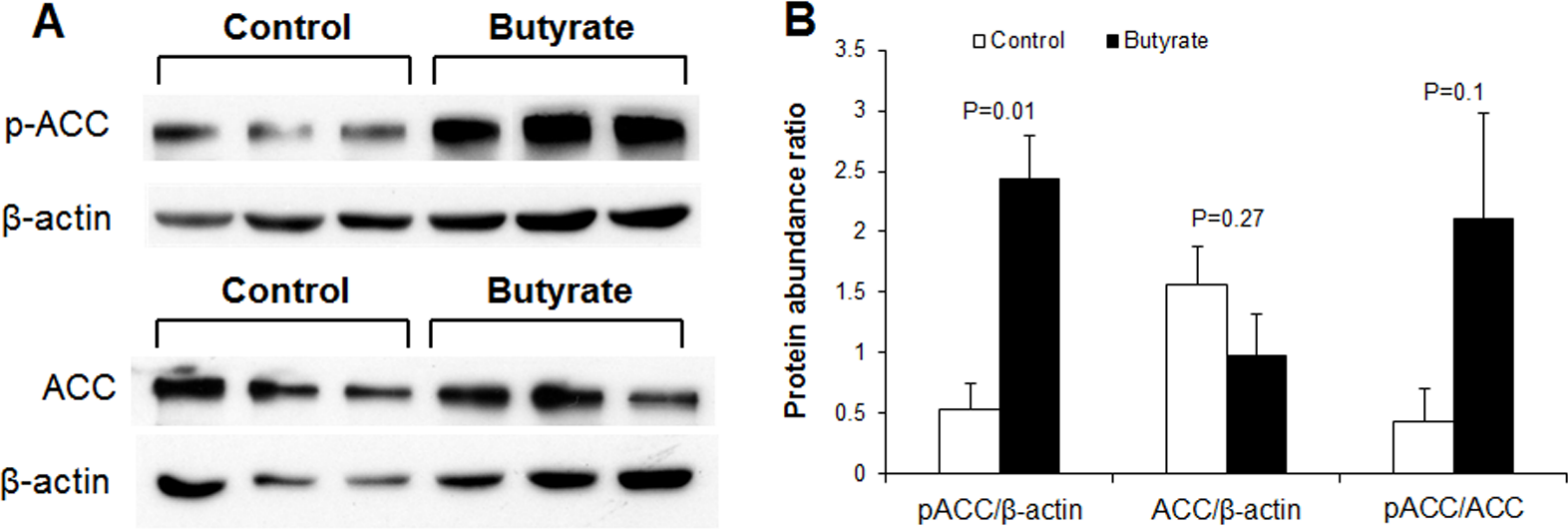
Effect of butyrate on ACC phosphorylation in adipocytes. (A) The representative immunoblotting analysis of ACC in adipocytes. (B) Quantification analysis of ACC in adipocytes. Preadipocytes were treated with butyrate at 1500 μM throughout the differentiation period (day 0 to 9). Cells were harvested at day 9 for immunoblotting. Data were analyzed by *t* test, and *P* values were shown on the bars (Control vs. Butyrate). Data are means ± SE (n = 4).

**Fig 8:**
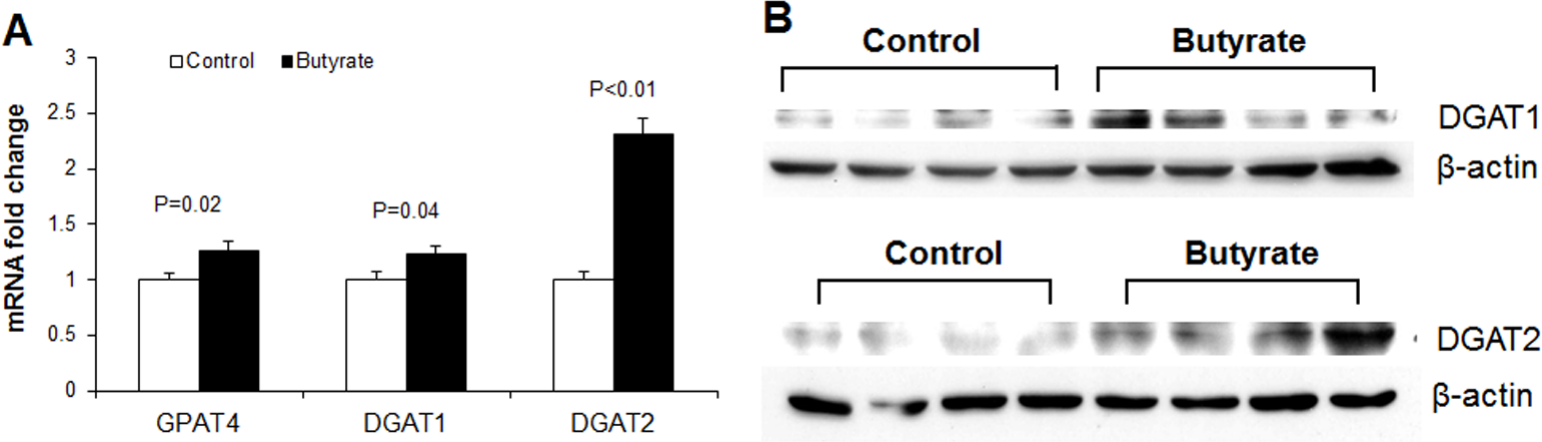
Effects of butyrate on expressions of triglyceride synthesis markers. (A) mRNA expression of triglyceride synthesis markers. Data were analyzed by *t* test, and *P* values were shown on the bars (Control vs. Butyrate). Data are means ± SE (n = 12). (B) Representative immunoblotting analysis of DGAT1 and DGAT2 in adipocytes. Preadipocytes were treated with butyrate at 1500 μM throughout the differentiation period (day 0 to 9). Cells were harvested at day 9 for real-time PCR and immunoblotting.

**Fig 9:**
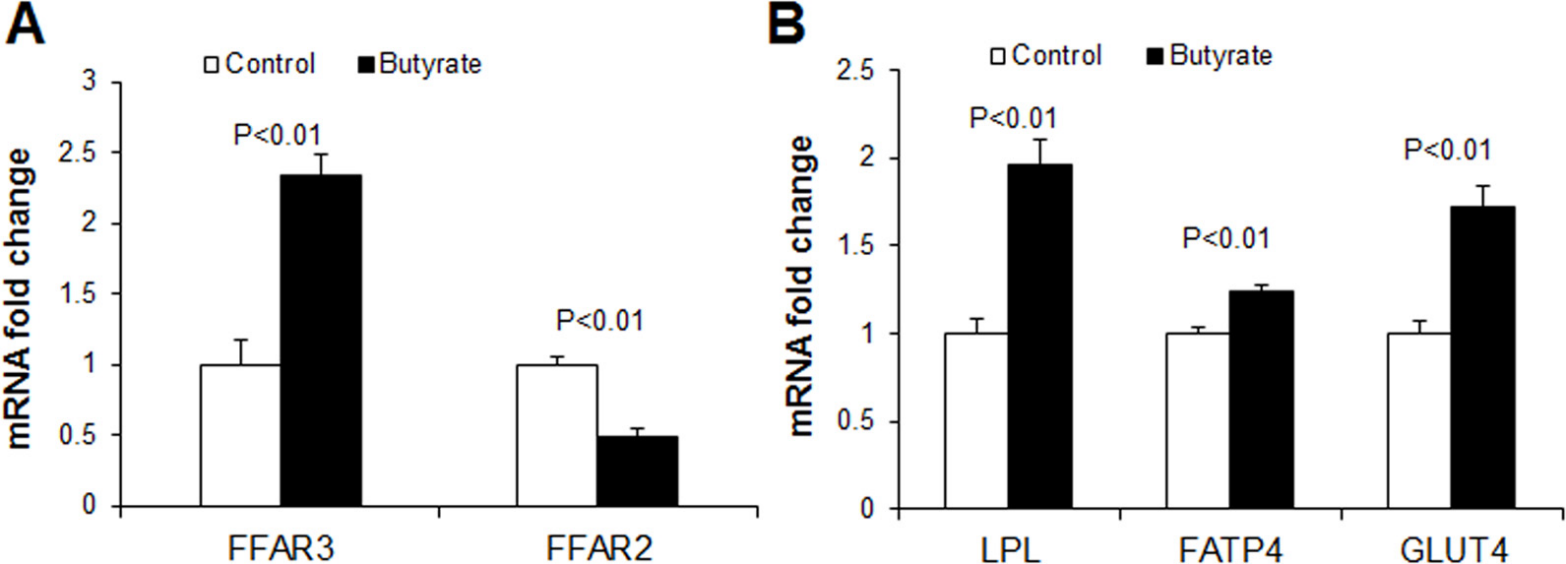
Effects of butyrate on mRNA expression of transporter proteins. (A) mRNA expression of short chain fatty acid receptors, FFAR3 and FFAR2. (B) mRNA expression of fatty acid and glucose uptake related lipase and transporter. Preadipocytes were treated with butyrate at 1500 μM throughout the differentiation period (day 0 to 9). Cells were harvested at day 9 for real-time PCR. Data were analyzed by *t* test, and *P* values were shown on the bars (Control vs. Butyrate). Data are means ± SE (n = 12).

To determine whether butyrate could influence markers of *de novo* fatty acid synthesis, we performed immunoblotting for acetyl-CoA carboxylase (ACC), the enzyme that catalyzes the first committed step of *de novo* fatty acid synthesis, carboxylation of acetyl-CoA which produces malonyl-CoA. Phosphorylation of ACC inactivates it, leading to suppression of *de novo* fatty acid synthesis. A marked increase in phospho-ACC abundance and phospho-ACC/ACC ratio was observed in butyrate-treated cells compared to control (Fig 7). To further clarify effect of butyrate on *de novo* fatty acid synthesis, we performed *de novo* lipogenesis assay in control and insulin-stimulated cells in the presence or absence of butyrate. Higher level of D-[^14^C]-glucose was incorporated into lipids in butyrate-treated cells, compared to control (P<0.01) (Fig 5B), suggesting that butyrate induces *de novo* fatty acid synthesis. Thus the increased phosphorylation of ACC by butyrate observed (Fig 7A) may have resulted from feedback inhibition of ACC. The increase in triglyceride synthesis in butyrate-treated cells leads to coordinate upregulation of expression of genes involved in fatty acid esterification. As confirmed with real-time PCR and immunoblotting, butyrate supplementation significantly increased expression of key enzymes involved in triglyceride synthesis (GPAT4, DGAT1 and DGAT2 mRNA expression) (Fig 8A). Butyrate-treated cells also had higher protein abundance of DGAT1 and DGAT2 compared to control (Fig 8B). The reaction catalyzed by DGAT is considered the final committed step in triglyceride synthesis [23]. Butyrate also significantly increased LPL, FATP and GLUT4 mRNA expression (Fig 9B), all of which are implicated in increased lipid storage. Thus butyrate action leads to coordinate upregulation of cellular pathways for increased triglyceride storage.

### Butyrate inhibits lipolysis

**Fig 10:**
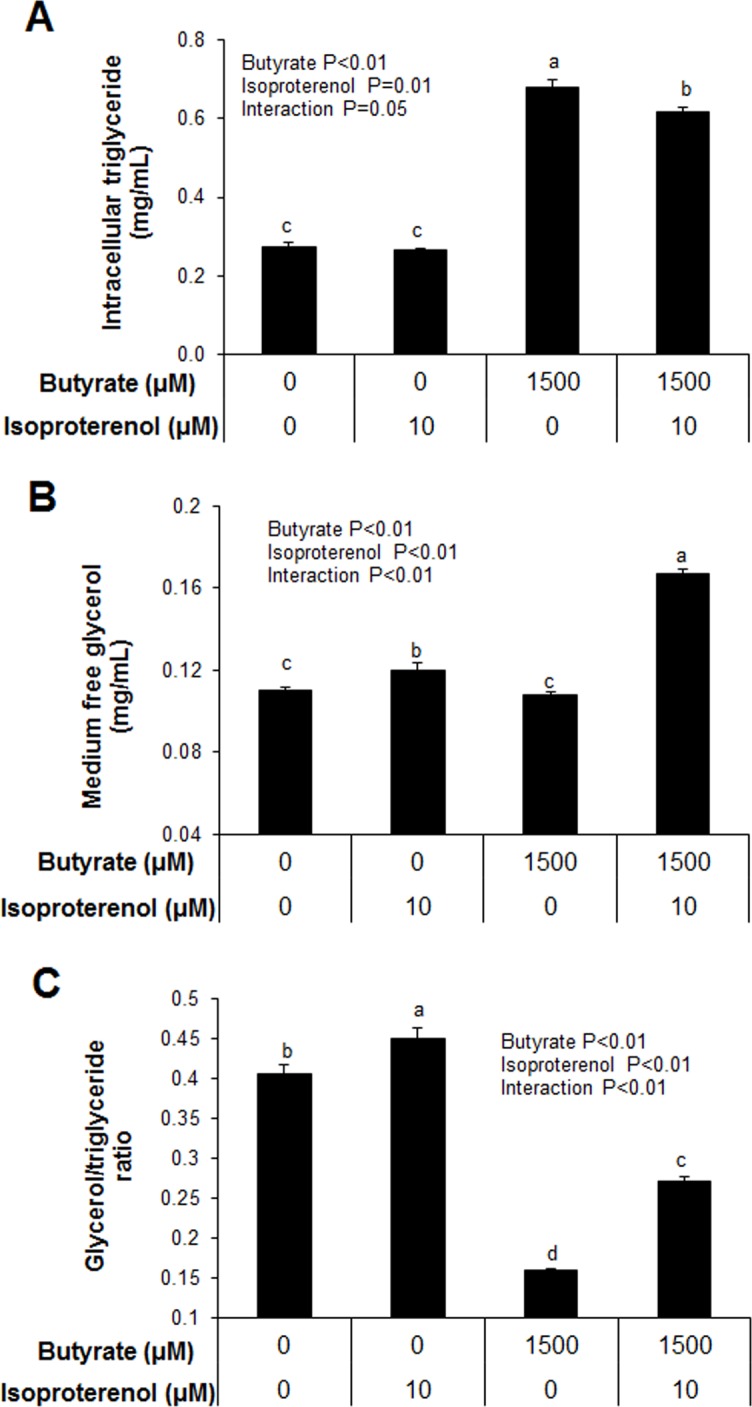
Effects of butyrate on intracellular triglyceride and free glycerol levels. (A) Intracellular triglyceride level. (B) Free glycerol level in the culture media. (C) Intracellular triglyceride to free glycerol ratio. Preadipocytes were treated with butyrate at 1500 μM throughout the differentiation period (day 0 to 9). Isoproterenol (ISO) at 10 μM was applied to adipocytes treated with/without butyrate for 2 hours before harvesting on day 9 of differentiation. Both cells and culture media were harvested for triglyceride and glycerol measurement. Data were analyzed by *two-way* ANOVA with Tukey multiple comparison test. Data are means ± SE (n = 8). Different letters on bars indicate significant differences (*P*<0.05).

To determine whether butyrate had effects on lipolysis, we measured intracellular triglyceride and free glycerol levels in the cell culture media in control (untreated) or isoproterenol-stimulated cells in the presence or absence of butyrate. As expected, butyrate-treated adipocytes had significantly higher intracellular triglyceride accumulation than control (P<0.01, Fig 10A). In butyrate-treated cells, isoproterenol significantly reduced intracellular triglyceride concentration (Fig 10A). In addition, isoproterenol increased medium glycerol in both control and butyrate-treated cells (Fig 10B). To determine the magnitude of lipolytic response in control and butyrate-treated cells relative to total intracellular triglyceride levels, we determined the ratio of media free glycerol and intracellular triglyceride ratio. The level of glycerol/triglyceride was lower in butyrate-treated cells (P<0.01) compared to control (Fig 10C), suggesting that butyrate countered both basal and isoproterenol-stimulated lipolysis.

It has previously been reported that the inhibitory effects of both acetate and propionate on lipolysis was via activation of FFAR2 [24, 25]. FFAR2-mediated inhibition of lipolysis is achieved through inactivation of (protein kinase A) PKA pathway via a pertussis toxin-sensitive G (G_i/o_) protein which inhibits adenylate and subsequently PKA and hormone sensitive lipase (HSL) [26]. Although both FFAR2 and FFAR3 are both G-protein coupled receptors for SCFAs and share most functions [5], butyrate has a higher affinity for FFAR3 compared to FFAR2 [27, 28]. We determined that butyrate-treated cells had a higher FFAR3 and lower FFAR2 expression (Fig 9A). The upregulation of FFAR3 by butyrate may be a possible mechanism by which it inhibited lipolysis.

### Effect of inhibition of peroxisomal and mitochondrial fatty acid oxidation on metabolic gene markers and triglyceride accumulation

**Fig 11:**
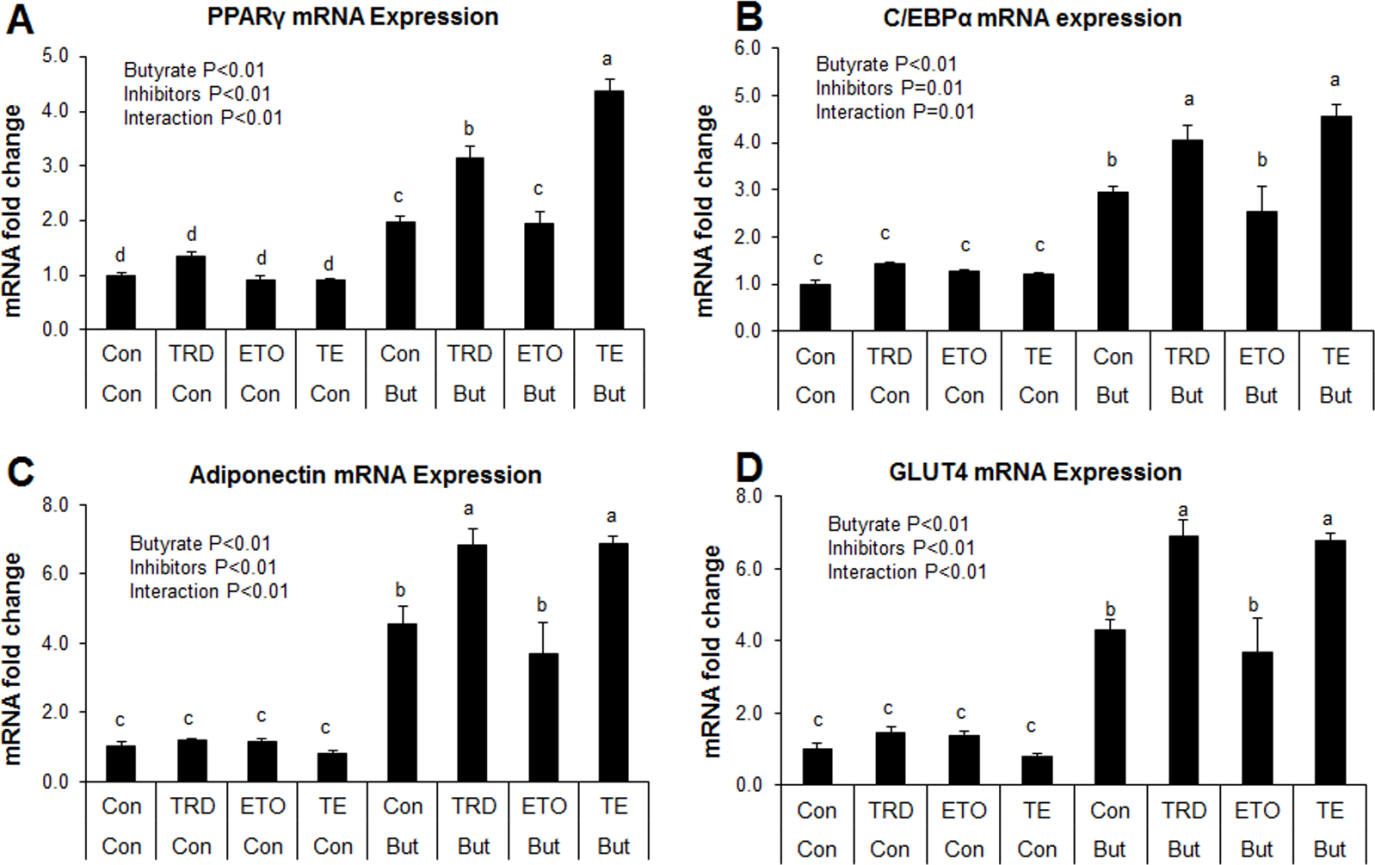
Interaction between butyrate and fatty acid oxidation inhibitors on expression of metabolic markers. Cells were treated with etomoxir (ETO) at 1 μM, thioridazine (TRD) at 10 μM or a combination of the two (TE). Inhibitors were applied with/without butyrate (But) at 1500 μM concentration throughout the differentiation period (day 0 to 9). Cells were harvested on day 9 for real-time PCR. Data were analyzed by *two-way* ANOVA with Tukey multiple comparison test. Data are means ± SE (n = 8). Different letters on bars indicate significant differences (*P*<0.05).

**Fig 12:**
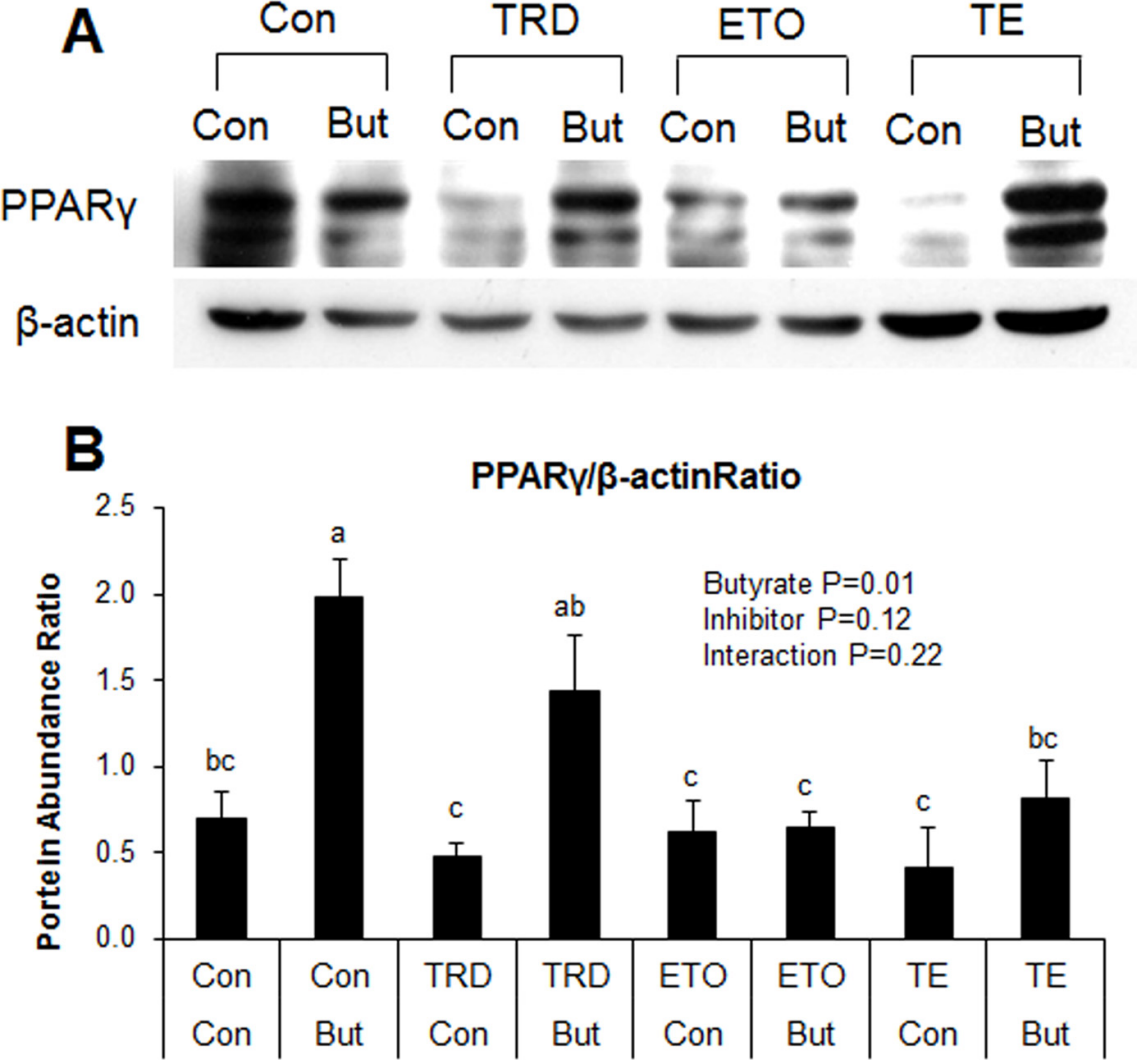
Interaction between butyrate and fatty acid oxidation inhibitors on PPARγ abundance. (A) Representative immunoblotting analysis of PPARγ in adipocytes. (B) Quantification analysis of PPARγ in adipocytes. Cells were treated with etomoxir (ETO) at 1 μM, thioridazine (TRD) at 10 μM or a combination of the two (TE). Inhibitors were applied with/without butyrate (But) at 1500 μM concentration throughout the differentiation period (day 0 to 9). Cells were harvested at day 9 for immunoblotting. Data were analyzed by *two-way* ANOVA with Tukey multiple comparison test. Data are means ± SE (n = 8). Different letters on bars indicate significant differences (*P*<0.05).

**Fig 13:**
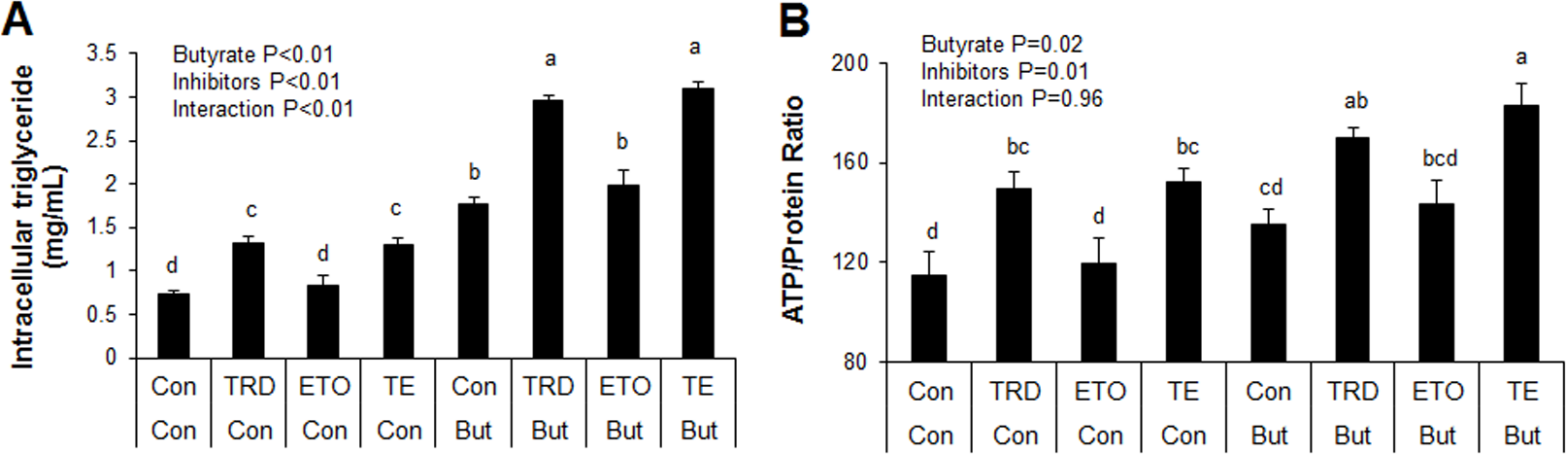
Interaction between butyrate and fatty acid oxidation inhibitors on intracellular triglyceride and ATP levels. (A) Intracellular triglyceride levels. (B) ATP level was normalized to total protein concentration. Cells were treated with etomoxir (ETO) at 1 μM, thioridazine (TRD) at 10 μM or a combination of the two (TE). Inhibitors were applied with/without butyrate (But) at 1500 μM throughout the differentiation period (day 0 to 9). Cells were harvested on day 9 for triglyceride and ATP determination assays. Data were analyzed by *two-way* ANOVA with Tukey multiple comparison test. Data are means ± SE (n = 8). Different letters on bars indicate significant differences (*P*<0.05).

Induction of ACO gene expression by butyrate suggests it may upregulate peroxisomal fatty acid oxidation. This is consistent with results from our previous *in vivo* study in which higher expression of ACO was found in the adipose and liver tissues of soluble fiber-fed pigs, which also had increased butyrate production in the hindgut, compared to animals fed insoluble fiber [3]. Therefore, these results suggest that butyrate could exert some of its effects via induction of fatty acid oxidation. To determine whether regulation of fatty acid oxidation pathways are important for butyrate effect, especially with regards to adipocyte differentiation and lipid accumulation, adipocytes were treated with either a peroxisomal (thioridazine, TRD) or mitochondrial (etomoxir, ETO) fatty acid oxidation inhibitors or a combination of the two (TE). As expected, butyrate significantly increased PPARγ and C/EBPα expressions (P<0.01). Additionally, both TRD and TE resulted in significantly higher PPARγ and C/EBPα expression compared to the ETO and control groups in butyrate-treated cells (P<0.01) (Fig 11). Furthermore, immunoblotting showed that butyrate significantly increased PPARγ protein abundance compared to control (Fig 12). Both TRD and TE supplementation enhanced butyrate-induced increase in PPARγ protein abundance (Fig 12). Butyrate significantly increased adiponectin and GLUT4 expression compared to control, while TRD and TE enhanced the effect of butyrate on the expression of these genes (Fig 11). In support of the gene expression and immunoblotting data, butyrate significantly increased TG (P<0.01) and ATP (P = 0.02) concentrations compared to control. In addition, TRD and TE supplementation additively increased the concentration of TG (P<0.01) and ATP (P = 0.02) with butyrate (Fig 13).

Taken together, these results indicate that butyrate significantly induces PPARγ, C/EBPα, adiponectin and GLUT4 mRNA expression and increases TG and ATP concentrations in adipocytes. The additive effects of the peroxisomal oxidation inhibitor, TRD, with butyrate on these responses indicate that butyrate may in fact exert some of its effects via inhibition of peroxisomal fatty acid oxidation. The fact that the mitochondrial oxidation inhibitor, ETO, had a very limited effect on these responses may indicate that butyrate effects may not be mediated via regulation mitochondrial fatty acid oxidation, perhaps because, compared to peroxisomal fatty acid oxidation, mitochondrial fatty acid oxidation is low in white adipocytes.

## Discussion

Short chain fatty acids, main products of microbial fermentation of dietary fiber in the large intestine, have been shown to have multiple beneficial effects on metabolism [5]. Primarily, SCFAs are used as energy source by enterocytes and for gluconeogenesis in ruminants, where they contribute approximately 70% of the daily caloric requirements, approximately 20–30% for several herbivorous animals, and approximately 10% for omnivorous species, such as humans and pigs [29]. Since pigs most closely resemble humans, primary pig adipocytes were used in our research to determine the roles of different SCFAs in adipocyte differentiation, triglyceride accumulation and adipokine expression. Compared to the two other SCFAs, acetate and propionate, butyrate shows greatest effects on the expression of gene markers, indicating that butyrate may have other unique properties different from acetate or propionate through which it is able to exert profound effects on adipocyte metabolism.

We demonstrate herein that butyrate supplementation leads to enhancement of adipogenesis, lipogenesis and adipokine expression. Butyrate effects appear to be partly mediated by activation of pathways involved in insulin sensitivity, as evidenced by the increased phosphorylation of AKT by butyrate. In addition, butyrate may promote triglyceride accumulation by coordinate regulation of expression of key enzymes of triglyceride synthesis, such as GPAT4, DGAT1 and DGAT2. Overall, the increased glucose uptake and *de novo* fatty acid synthesis in butyrate-treated cells may drive substrates towards increased fatty acid esterification and hence triglyceride synthesis.

Furthermore, enhancement of adipocyte differentiation by butyrate, as evidenced by its upregulation of SREBP-1c, C/EBPα/β and PPARγ expression may potentially result from its inhibition of histone deacetylation [30, 31]. Transcription initiation and elongation requires histone acetylation to open chromatin DNA for contacts transcription factors. Histone deacetylase inhibits gene transcription through deacetylation of histone proteins [32, 33]. Butyrate, a histone deacetylase inhibitor, suppresses histone deacetylase and consequently enhances histone acetylation [34, 35]. It has recently being shown that butyrate increases total acetylated histones at the promoter of C/EBPα in porcine adipocytes [31], suggesting that chromatin modification occurs on the promoters of transcription factors implicated in adipocyte differentiation in the presence of butyrate.

The increased adiponectin mRNA expression and protein secretion by butyrate may have resulted from increased adipocyte differentiation. Adiponectin is a hormone secreted by adipocytes that exerts multiple functions in regulating energy homeostasis and glucose and lipid metabolism [22]. Adiponectin triggers AMPK phosphorylation leading to increased energy expenditure and decreased fatty acid synthesis. Adiponectin also improves insulin sensitivity through inhibiting inflammatory signaling [36]. In this study, butyrate supplementation leads to AMPK activation. SCFAs have been reported to induce AMPK activation, but the mechanism is still unclear [5]. Our study suggests that butyrate may have activated AMPK through increased adiponectin secretion, and that butyrate supplementation may be a strategy to increase adiponectin production. In 3T3-L1 murine adipocytes, activation of AMPK inhibits adipocyte differentiation [37], which would seem contrary to the present findings. However, it has been reported that activation of AMPK did not block butyrate-induced C/EBPα/β and PPARγ expression in porcine adipocytes, suggesting that butyrate may stimulate adipocyte differentiation independent of AMPK-mediated pathways [31]. AMP-activated protein kinase (AMPK) plays an important role in regulating fatty acid metabolism in various tissues. In the liver and muscle, butyrate increases phosphorylation of AMPK [8]. Activation of AMPK is also linked to increased expression of peroxisome proliferator-activated receptor gamma coactivator (PGC-1α), a known inducer of mitochondrial biogenesis [38]. As a result, mitochondrial fatty acid oxidation is induced in both tissues and *de novo* fatty acid synthesis is inhibited in the liver. In addition, SCFA-induced AMPK activation leads to increases in PGC-1α and uncoupling protein (UCP)-1 protein expression in brown adipose tissue, thereby increasing fatty acid oxidation and thermogenesis [5, 8]. Although it may appear that butyrate increases peroxisomal fatty acid oxidation because it induces ACO gene expression, experiments with the peroxisomal fatty acid inhibitor, TRD, provide evidence to the contrary. This is because the effect of butyrate on lipid storage and gene expression is further amplified in the presence of TRD, whereas TRD is ineffective in control cells, suggesting butyrate may exert part of its effects through interaction with peroxisomal fatty acid oxidation pathway. The exact nature of this interaction remains unclear at this point. Unlike TRD, inhibition of mitochondria fatty acid oxidation with etomoxir has no significant effect on butyrate action. This is consistent with the limited number of mitochondria and limited contribution of mitochondria oxidation to the overall fatty acid oxidation in white adipocytes. These results demonstrate that effects of butyrate on fatty acid oxidation in the liver, muscle and brown adipose tissue cannot be extrapolated to white adipose tissue. Although AMPK is activated by phosphorylation in adipocytes, this effect likely does not lead to increased mitochondria biogenesis and mitochondria fatty acid oxidation.

We also demonstrate an inhibitory effect of butyrate on lipolysis. Butyrate induced inhibition of lipolysis is likely through activation of FFAR3, because butyrate treatment is accompanied by increased expression of FFAR3. Studies have shown that acetate and propionate inhibit lipolysis via FFAR2 activation [24, 25]. FFAR2 and FFAR3 belong to G protein-coupled receptors family and are identified as SCFA receptors. FFAR2 and FFAR3 have differences in affinity for SCFAs, but similar physiological functions. FFAR3 has a higher preference for butyrate, whereas FFAR2 has a higher affinity for acetate and propionate [27, 28]. Both FFAR3 and FFAR2 can couple to pertussis toxin-sensitive (G_i/o_) G protein [27, 28]. FFAR2 also couples to pertussis toxin-insensitive (G_q_) G proteins [27, 28]; however, approximately 70% of FFAR2 signals are transduced through the G_i/o_ pathway [39]. Because of the higher affinity of butyrate for FFAR3 than FFAR2, we hypothesize that butyrate may exert effect on inhibition of lipolysis possibly through binding to FFAR3. The inhibition of lipolysis, perhaps mediated by FFAR3, is most likely effected by inactivation of hormone-sensitive lipase (HSL) through inactivation of the adenylate cyclase (AC) pathway. The mechanism of inhibition of lipolysis by butyrate may follow the classical mechanism in which binding of butyrate to FFAR3 triggers dissociation and activation of the pertussis toxin-sensitive (G_i/o_) G protein, which then inhibits adenylate cyclase (AC) and thereby reduces cAMP production. Reduction of cAMP level results in low PKA activity, and reduced activation of HSL, a key enzyme controlling lipolysis in adipocytes [5, 26].

**Fig 14:**
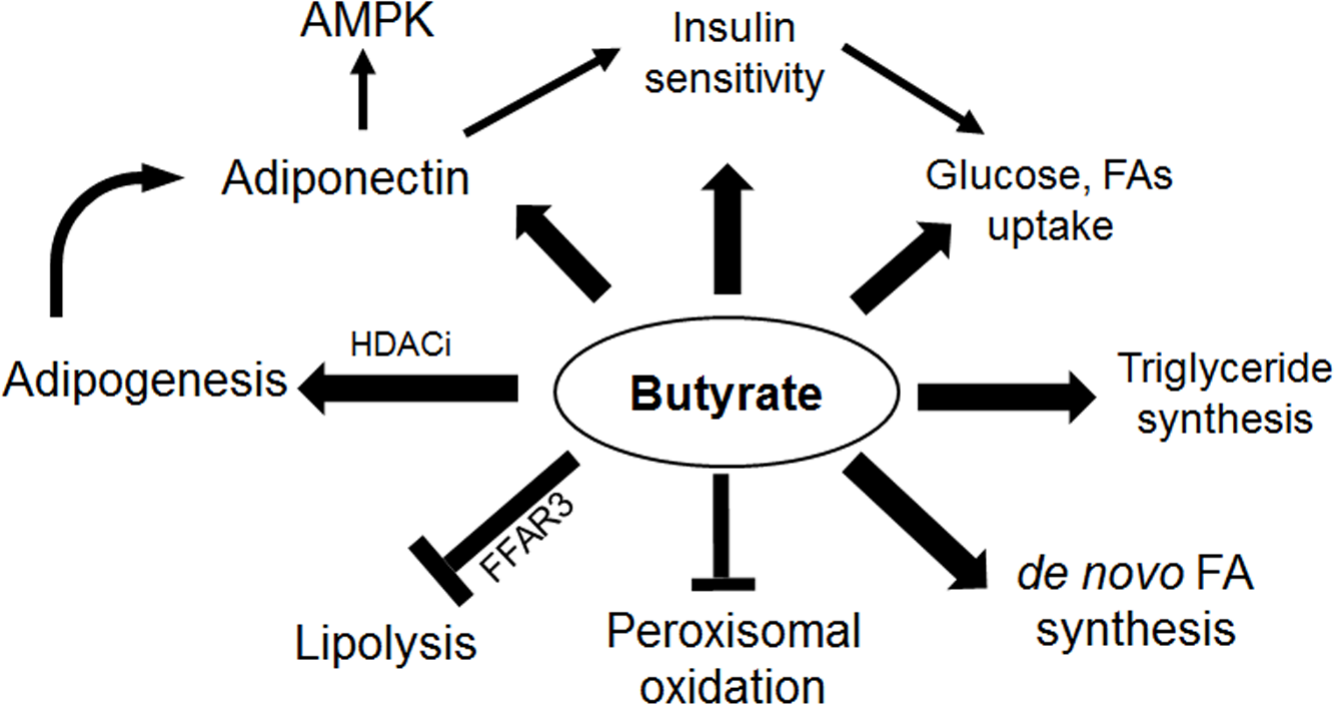
Schematic summary of the effects of butyrate on adipogenesis and lipid metabolism. Lines with arrowhead represent upregulation of activity, protein content, and/or expression. Lines without arrowhead denote inhibition. Butyrate supplementation leads to induction of adipogenesis, which results in increased adiponectin secretion. Adiponectin activates the AKT pathway, thereby increasing insulin sensitivity. Adiponectin also leads to activation of AMPK. Butyrate increases intracellular triglyceride accumulation through inhibition of lipolysis and enhancement of triglyceride synthesis. Butyrate also inhibits peroxisomal fatty acid oxidation, leading to accumulation of LCFAs, which may also account for feedback inhibition of *de novo* fatty acid synthesis through increased ACC phosphorylation.

In summary, we have shown that butyrate may exert direct effects on adipocytes in the regulation lipid storage through inhibition of lipolysis and enhancement of fatty acid synthesis and esterification (Fig 14). Butyrate effects could be partly mediated through interaction with peroxisomal fatty acid oxidation, because the peroxisomal inhibitor, TRD, enhanced butyrate effects. However, the nature of this interaction is still unclear. Inhibition of lipolysis, stimulation of glucose uptake and induction of triglyceride synthesis by butyrate suggests its potential utility in preventing, or reversing hyperglycemia and hyperlipidemia. This study, in addition to previous evidence of butyrate effect in increasing fatty acid oxidation in the liver, muscle and brown adipose tissue, suggests that butyrate may also target white adipocytes to combat hyperlipidemia and enhance insulin sensitivity.

## Supporting Information

S1 Table List of real-time PCR primers. (DOCX)
