# Supplementary material for: Mechanism of Butyrate Stimulation of Triglyceride Storage and Adipokine Expression during Adipogenic Differentiation of Porcine Stromovascular Cells
Source: PLoS One. 2015 Dec 29;10(12):e0145940. doi: 10.1371/journal.pone.0145940 (PMC4694642; doi:10.1371/journal.pone.0145940)
Supplement: S1 Table — (DOCX) [file pone.0145940.s001.docx]

**S1 Table. List of real-time PCR primers**

| Gene | Forward | Reverse |
| --- | --- | --- |
| 18S | 5'- ATC CCT GAG AAG TTC CAG CA -3' | 5'- CCT CCT GGT GAG GTC GAT GT -3' |
| ACO | 5'- GGA CGG CAG TCC AGA GAA TA -3' | 5'- GGT GGC GCT CTT CTT AAC AG -3' |
| CPT1α | 5'- GTC AGC GTA GCA AGT GGA CA -3' | 5'- GTG ACG TTA CAT CCC CTG CT -3' |
| SREBP-1c | 5'- ACC GCT CTT CCA TCA ATG AC -3' | 5'- AAT GTA GTC GAT GGC CTT GC -3' |
| FAS | 5'- AGT TTG TGA TGG AGA ACA CGG CCT-3' | 5'- TGT TCA CAC GTG GTG CAA GGG TTA -3' |
| Adiponectin | 5'- TGG AGA AAG CGC CTA TGT CT -3' | 5'- TTT GCC AGT GGT GAC ATC AT -3' |
| Leptin | 5'- TTG GCC CTA TCT GTC CTA CG -3' | 5'- GTG ACC CTC TGT TTG GAG GA -3' |
| GLUT4 | 5'- GAA GGA AGA AGG CAA TGC TG -3' | 5'- GAG GAA CCG TCC AAG AAT GA -3' |
| PPARγ | 5'- GCC CTT CAC CAC TGT TGA TT -3' | 5'- GTT GGA AGG CTC TTC GTG AG -3' |
| C/EBPα | 5'- TGG ACA AGA ACA GCA ACG AG -3' | 5'- TTG TCA CTG GTC AGC TCC AG -3' |
| C/EBPβ | 5'- GCT TGA ACA AGT TCC GCA GG -3' | 5'- CAA GAA GAC GGT GGA CAA GC -3' |
| RXRα | 5'- GCA TCC AGA AGA ACA TGG TGT -3' | 5'- CCT GCT TGG CGA ACT CCA CAG T -3' |
| LPL | 5'- ATT CAC CAG AGG GTC ACC TG -3' | 5'- AGC CCT TTC TCA AAG GCT TC -3' |
| FATP4 | 5'- CAT TGT GGC TCA GCA GGT TA -3' | 5'- CAG GCT AGG GGT CAA ATC AA -3' |
| FFAR3 | 5'- AGG CAA GTG GAC CCC GCT GA -3' | 5'- GCA GCC CCA CGA GGA ACG TC -3' |
| FFAR2 | 5'- GGT AAA GGC CGG ACC CTG GAG -3' | 5'- GGA GGT TGG CTG GGA GAC CAG T -3' |
| GPAT4 | 5'- GCT GAA GAT CTT TGC GTG GG -3' | 5'- GCT TGT ACA GCT GGT GGT TCT -3' |
| DGAT1 | 5'- CCA GCA GAG GGT TCA AGA CA -3' | 5'- GAC TAG CTG CCT CAT CCA GG -3' |
| DGAT2 | 5'- CAC CAT CTC CTT CGG GGA GA -3' | 5'- CTT GGA GTA GGG CAT GAG CC -3' |
